# Evaluation of extracorporeal cardiopulmonary resuscitation eligibility criteria for out-of-hospital cardiac arrest patients

**DOI:** 10.1186/s13104-021-05564-1

**Published:** 2021-04-15

**Authors:** Brendan Lee, Adam Clay, Eric Sy

**Affiliations:** 1 College of Medicine, University of Saskatchewan, Regina General Hospital, 1440 - 14th Avenue, Regina, SK S4P 0W5 Canada; 2 Research Department, Saskatchewan Health Authority, Wascana Rehabilitation Centre, 2180 - 23 Ave, Regina, SK S4S 0A5 Canada; 3 Department of Academic Family Medicine, University of Saskatchewan, 1621 Albert St #172, Regina, SK S4P 2S5 Canada; 4 Department of Critical Care, Regina General Hospital, 1440–14th Avenue, Regina, SK S4P 0W5 Canada

**Keywords:** Extracorporeal membrane oxygenation (ECMO), Cardiopulmonary resuscitation (CPR), Out-of-hospital cardiac arrest (OHCA), Resuscitation

## Abstract

**Objectives:**

To evaluate the number of out-of-hospital cardiac arrest (OHCA) patients eligible for extracorporeal cardiopulmonary resuscitation (ECPR) in Saskatchewan and their clinical outcomes, including survival and neurological outcomes at discharge. ECPR eligibility was assessed, using clinical criteria from the University of British Columbia (UBC, Canada), University of Michigan (UM, United States), University of California (UC, United States) and a restrictive ECPR criteria.

**Results:**

We performed a retrospective cohort study of 200 OHCA patients (August 1, 2017-May 31, 2019) in Regina, Saskatchewan. Sixty-one (30%) were female, the median age was 64 years (interquartile range [IQR], 52–78), the median CPR duration was 30 min (IQR 12–47), and 20% survived to discharge. Two (1%) patients received ECPR but did not meet any ECPR criteria. Nineteen (10%), thirty (15%), twenty-two (11%), and seven (4%) patients were ECPR-eligible, using the UBC, UM, UC, and restrictive criteria. However, none of these patients had received ECPR. Only two (11%), two (7%), two (9%), and one (14%) of these patient(s) survived to discharge, respectively. Neurological outcomes were unfavourable among all ECPR-eligible patients. Future study at our centre will be necessary on how to implement ECPR program to further improve these outcomes.

**Supplementary Information:**

The online version contains supplementary material available at 10.1186/s13104-021-05564-1.

## Introduction

There has been increasing interest in the use of extracorporeal cardiopulmonary resuscitation (ECPR) to improve outcomes in out-of-hospital cardiac arrest (OHCA) patients. Observational studies have demonstrated improved outcomes for OHCA patients who receive ECPR versus traditional resuscitation, including increased survival to hospital discharge and improved neurological outcomes [1–3]. Recently, a randomized trial of ECPR-facilitated resuscitation (ARREST trial) demonstrated improved survival to hospital discharge with ECPR compared to standard advanced cardiovascular life support [4]. However, ECPR is resource intensive, making the establishment of programs difficult [1]. In 2017, a Canadian ECPR working group was formed to identify knowledge gaps, determine ECPR capacity, perform economic analyses, build a dataset for research, and develop evidence-based eligibility criteria [6].

Many centres and programs have developed different eligibility criteria for ECPR. In the literature, we had identified three main eligibility criteria, using pre-existing ECPR eligibility criteria developed from three different ECPR programs and/or clinical trials from the University of British Columbia (UBC), University of Michigan (UM), and University of California (UC) [8–11]. These eligibility criteria are described further in Additional file 1: Table S1. The UBC criteria was previously described by Grunau et al. and is currently being used in an ongoing ECPR trial (NCT02832752) [10]. The UM criteria is currently being used in an ECPR trial (NCT03065647) [8]. The UC criteria was previously described by Bellezzo et al. [9].

Non-shockable rhythms (pulseless electrical activity [PEA] and asystole) have been associated with poor outcomes and generally has been considered as an exclusion to participation in major ECPR trials [1, 10, 12, 13]. Prior studies identified the presence of major comorbidities (i.e. chronic obstructive pulmonary disease, congestive heart failure, malignancy, pre-existing neurological deficits, renal failure requiring dialysis, and cirrhosis) as predictors of poor outcome following OHCA [10, 13].

The objectives of this study were to (a) determine what proportion of OHCA patients in a tertiary care institution in Saskatchewan would meet different ECPR criteria and (b) evaluate the outcomes of ECPR eligible OHCA patients.

## Main text

### Methods

We conducted a retrospective cohort study of consecutive OHCA patients presenting to the Regina General Hospital emergency department (ED) between August 1, 2017 and May 31, 2019 (REB-18–28). Regina General Hospital is a 468-bed tertiary care teaching hospital with 31 funded intensive care unit (ICU) and coronary care unit beds. The hospital is extracorporeal membrane oxygenation (ECMO)-capable and is staffed by board-certified emergency medicine physicians, cardiologists, intensivists, cardiothoracic surgeons, and perfusionists; however, there is no formal ECPR program. Patients were included in our study review for ECPR eligibility, if they were ≥ 18 years old and had a primary diagnosis of OHCA. In-hospital cardiac arrests were excluded.

From the included patients, we collected demographic, clinical and laboratory data, including age, gender, comorbidities, previous cardiac history, and arrest details to determine whether or not they met the established ECPR eligibility criteria. Neurological outcomes were evaluated using the Cerebral Performance Category scale (CPC) [7]. All data was collected and stored in REDCap (Vanderbilt University, United States). All pre-hospital data was collected through emergency medical services (EMS) report sheets. Hospital data was collected through the electronic medical record or paper charts. Missing data were recorded as unknown. However, if the missing data was critical to the analysis of patient outcomes, inclusion and/or exclusion criteria of the study, then the patient was excluded. Only two patients were excluded for insufficient information.

The primary outcome of this study was to determine the proportion of patients meeting the previously described ECPR eligibility criteria. We also developed a more restrictive eligibility criteria, based on prior described criteria to further assess the feasibility of initiating ECPR in centers with limited resources. In the restrictive eligibility criteria, patients were eligible to receive ECPR if they met the following criteria: (a) 18–60 years of age, (b) initial presenting rhythm was shockable, (c) at least 15 min of CPR had elapsed without return of spontaneous circulation (ROSC) (d) the cause of arrest was presumed to be of cardiac origin, and (e) no significant pre-existing medical comorbidities. Secondary outcomes included the comparison of in-hospital mortality, hospital and ICU length of stay, and neurologic outcome at discharge between ECPR eligible and ECPR-ineligible patients. Neurological outcomes were evaluated using the Cerebral Performance Category (CPC) at hospital discharge.

All analyses were performed using Statistical Package for Social Sciences (SPSS) version 22 (IBM) or Stata/MP 15.1 (StataCorp). Categorical variables were described as counts (proportions). Continuous variables were described as means (standard deviation [SD]) or medians (interquartile range [IQR]) when appropriate, following testing for normality using skewness and kurtosis. Group comparisons and statistical testing were performed between ECPR-eligible patients (for any criteria) versus ECPR-ineligible patients (i.e., no ECPR criteria met). Categorical variables were compared primarily using χ^2^_,_ or Fisher’s exact test when there were few observations (i.e., < 10 per group). Continuous variables were compared using Student’s t-test, or Wilcoxon rank sum test for not normally distributed data. A two-sided p-value < 0.05 was considered statistically significant for all analyses.

### Results

**Fig. 1:**
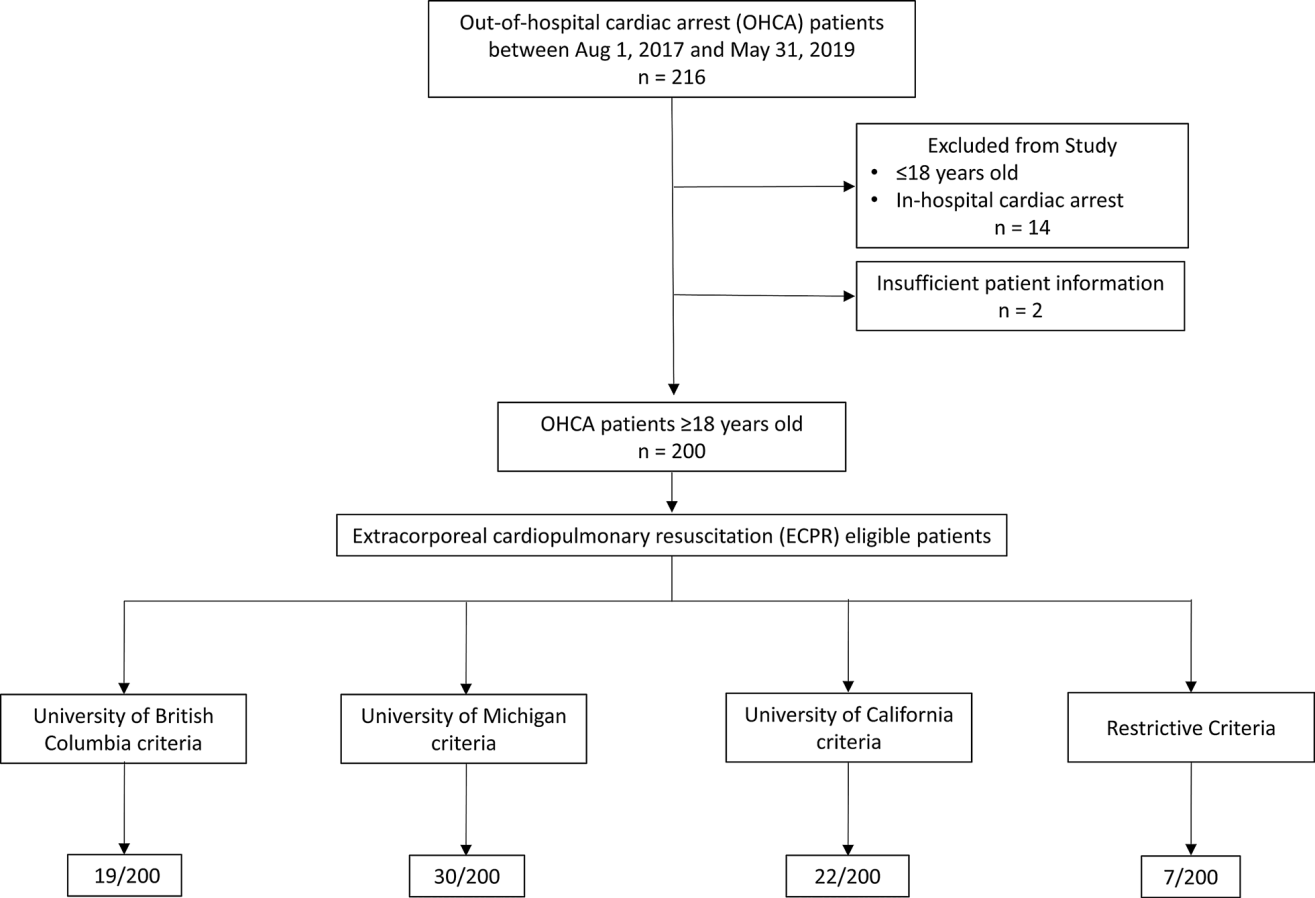
Flow diagram of the study

We included 200 patients in this study from 216 potential patients (Fig. 1). Patient demographics and cardiac arrest characteristics are presented in Table 1, Additional file 1: Table S2, S3. In this study, 139 (70%) patients were male, the median age was 64 (IQR, 52–78). Among survivors (n = 40, 20% of all patients), the mean age was 57 (IQR 44–69) and 31 (78%) were male. The most common presenting cardiac rhythm was PEA in 86 (44%) cases, ventricular fibrillation in 54 (28%) cases, and asystole in 45 (23%) of cases.

**Table 1:** Interventions and outcomes of all out-of-hospital cardiac arrest patients

Patient characteristic		Any ECPR criteria met (N = 35)	No ECPR criteria met (N = 165)	Total (n = 200)	p-value	Number with missing data
ICU or CCU admission, N (%)		10 (29)	63 (38)	73 (37)	0.27	1
Interventions, N (%)	Coronary angiography	5 (14)	28 (17)	33 (17)	0.70	–
	PCI	5 (14)	28 (17)	17 (9)	0.99	–
	CABG	1 (3)	4 (2)	5 (3)	0.88	–
	ECPR	0 (0)	2 (1)	2 (1)	0.51	2
Duration of CPR performed, median mins (IQR)		42 (30–60)	25 (10–45)	30 (12–47)	< 0.001	54
Complications, N (%)	Circulatory shock	5 (14)	44 (27)	49 (25)	0.12	–
	Need for RRT	2 (6)	12 (7)	14 (7)	0.74	–
	Stroke	0 (0)	3 (2)	3 (2)	0.42	1
	Intracerebral hemorrhage	0 (0)	2 (1)	2 (1)	0.51	–
Hospital length of stay, median days (IQR)		0 (0–2)	0 (0–5)	0 (0–4)	0.09	2
ICU length of stay, median days (IQR)		0 (0–1)	0 (0–2)	0 (0–2)	0.32	2
Cerebral Performance Category at discharge, N (%)	1	0 (0)	18 (11)	18 (9)	0.16	–
	2	2 (6)	13 (8)	15 (8)		
	3	0 (0)	6 (4)	6 (3)		
	4	0 (0)	1 (1)	1 (0)		
	5	33 (94)	127 (77)	160 (80)		
In-hospital mortality, N (%)		33 (94)	127 (77)	160 (80)	0.02	–

Coronary artery bypass grafting (CABG), extracorporeal cardiopulmonary resuscitation (ECPR), Glasgow Coma Scale (GCS), Interquartile range (IQR), number (N), out-of-hospital cardiac arrest (OHCA), percutaneous intervention (PCI), pulseless electrical activity (PEA), return of spontaneous circulation (ROSC), renal replacement therapy (RRT), standard deviation (SD), ventricular fibrillation (VF), ventricular tachycardia (VT)

Categorical variables were compared primarily using χ^2^, or Fisher’s exact test when there were few observations (i.e., < 10 per group). Continuous variables were compared using Student’s t-test, or Wilcoxon rank sum test for not normally distributed data. A two-sided p-value < 0.05 was considered statistically significant for all analyses

The median CPC score for all patients was 5 (IQR 5–5). In-hospital mortality was 80%, with 40 (20%) patients surviving to hospital discharge. In survivors, the most common etiology of arrest was ischemic heart disease for 17 (43%) patients and drug overdose for 10 (25%). Among survivors, the median CPC score was 2 (IQR 1–2), the median ICU length of stay was 3 (0–5) days, and the median hospital length of stay was 9 (IQR 5–16) days. For non-survivors, the most common etiology of cardiac arrest was ischemic heart disease in 76 (48%).

Between the four different criteria, 19 (10%) met the UBC criteria, 30 (15%) met the UM criteria, 22 (11%) met the UC criteria, and 7 (4%) met the restrictive ECPR eligibility criteria (Table 2). Of the two patients who had initiation of ECPR, none of these patients met any of the four eligibility criteria. The median CPC score for ECPR-eligible patients was 5 (IQR 5–5), demonstrating overall poor neurological outcomes, in all four criteria. Only 2 (11%), 2 (7%), 2 (9%), and 1 (14%) of patients survived to hospital discharge among the UBC, UM, UC, and restrictive ECPR criteria, respectively.

**Table 2:** Characteristics and outcomes of out-of-hospital cardiac arrest patients eligible for ECPR

		UBC Criteria (N = 19)	UM Criteria (N = 30)	UC Criteria (N = 22)	Restrictive Criteria (N = 7)	Non-eligible for any of the criteria (N = 165)
Criteria Met*, N (%)		19 (10)	30 (15)	22 (11)	7 (4)	165 (83)
Age, median years (IQR)		51 (43–60)	56 (46–62)	52 (45–61)	43 (35–53)	66 (54–80)
Male gender, N (%)		16 (84)	24 (80)	18 (82)	5 (71)	111 (67)
Charlson Comorbidity Index, median score (IQR)		0 (0–0)	0 (0–1)	0 (0–1)	0 (0–0)	1 (0–3)
APACHE II score, median score (IQR)		33 (27–39)	33 (28–37)	33 (27–37)	28 (23–36)	32 (27–37)
Duration of CPR performed, median mins (IQR)		45 (37–60)	43 (30–60)	43 (20–60)	43 (30–60)	25 (10–45)
ICU or CCU admission, N (%)		4 (21)	6 (20)	7 (32)	3 (43)	63 (38)
Hospital length of stay, median days (IQR)		0 (0–1)	0 (0–3)	0 (0–1)	0 (0–5)	0 (0–5)
ICU length of stay, median days (IQR)		0 (0–0)	0 (0–2)	0 (0–0)	0 (0–5)	0 (0–2)
Cerebral performance category at discharge, median (IQR)		5 (5–5)	5 (5–5)	5 (5–5)	5 (5–5)	5 (5–5)
Cerebral performance category at discharge, N (%)	1	0 (0)	0 (0)	0 (0)	0 (0)	18 (11)
	2	2 (11)	2 (7)	2 (9)	1 (14)	13 (8)
	3	0 (0)	0 (0)	0 (0)	0 (0)	6 (4)
	4	0 (0)	0 (0)	0 (0)	0 (0)	1 (1)
	5	17 (89)	28 (93)	20 (91)	6 (86)	127 (77)
Survival to hospital discharge, N (%)		2 (11)	2 (7)	2 (9)	1 (14)	38 (23)

^*^Of total number of patients in cohort

Cardiopulmonary resuscitation (CPR), coronary care unit (CCU), intensive care unit (ICU), interquartile range (IQR), number (N), University of British Columbia (UBC), University of California (UC), University of Michigan (UM)

Patients eligible for ECPR were younger (median age 56 years versus 66 years, p = 0.003), had fewer comorbidities (p < 0.001), and were more likely to have bystander witnessed arrest (p = 0.03) than ineligible patients. Additionally, they had longer duration of CPR (median 42 min versus 25 min, p < 0.001), higher initial lactate (p < 0.001), and lower initial arterial blood pH (p = 0.01), compared to ineligible patients. There were no significant differences in the initial cardiac rhythm (p = 0.49) or cause of arrest (p = 0.48) between groups. Hospital length of stay and ICU were similar between both groups. In-hospital mortality was higher among ECPR-eligible patients compared to ineligible patients (94% versus 77%, p = 0.02).

### Discussion

In this study, approximately 4% to 15% of OHCA patients met the eligibility criteria for ECPR, depending on the eligibility criteria used. While 20% of all OHCA patients survived to hospital discharge, only 7–14% of OHCA patients eligible for ECPR had survived to hospital discharge. In this study, only 2 (1%) of patients had initiation of ECPR. However, none of the patients who received ECPR had met any established ECPR eligibility criteria. In comparison, approximately 5 to 15 patients per year at this study’s institution would have been eligible to ECPR depending on the eligibility criteria used.

There have been several recent Canadian studies, evaluating the number of patients eligible for ECPR [10, 15, 16]. Grunau et al. found that 10.2% of OHCA patients presenting to Vancouver EDs were eligible for ECPR [10]. In Ottawa, a similar study identified that 6–15% of their OHCA patients were eligible for ECPR [16]. A retrospective study conducted in Saskatoon in 2016 found that 14% of non-survivors of OHCA represented suitable candidates for ECPR [9, 15]. In New Brunswick, Rollo et al. determined that approximately 5 patients annually would be eligible for ECPR at their hospital [18]. In Manitoba, Parr et al. reported that ECPR was a feasible intervention to support cardiac catherization laboratory patients in cardiac arrest or cardiogenic shock, with favorable 30-day (47%) and 1-year survival (44%) [17].

However, the establishment of ECPR programs must be tempered by proof of efficacy. Various observational studies have demonstrated improved outcomes (increased survival to hospital discharge and favourable neurological outcomes) for cardiac arrest patients who receive ECPR versus traditional resuscitation [9, 10, 19]. Yet, a systematic review by Holmberg et al. found that the certainty of evidence still remains very low and there was critical risk of bias [20]. A recent randomized trial has supported the use of ECPR-assisted resuscitation, but had an overall small sample size [4]. Ongoing randomized clinical trials (NCT02832752, NCT03065647, and NCT03101787) may provide higher quality evidence to answer this question.

Despite this, there is significant interest in establishing ECPR programs. In a survey of United States centres that submitted ECPR cases to the Extracorporeal Life Support Registry in 2016, there were 36 centres that had an ECPR program, of which 65% of programs were < 5 years old and 60% of programs had performed ≤ 3 cases per year [21]. Newer programs or smaller centres with less ECMO volume or experience may have worse outcomes [22]. In this study, four different eligibility criteria, varying in their degree of inclusivity, were evaluated to determine the number of potential annual ECPR cases. The goal of developing ECPR criteria is to balance the development of clinical expertise to optimize outcomes with the sustainability of such a program, taking into account the available resources and personnel [23]. Human resources, particularly trained perfusionists or ECMO specialists, remains a large barrier for implementation of ECMO.

Timeliness of ECPR is another important consideration when developing a program. Prior studies demonstrated a median time of initiation of ECPR of one hour [20, 24, 25]. However, prolonged low-flow duration (time from initiation of CPR to initiation of ECPR) has been associated with worse neurological outcomes [25]. Successful ECPR programs would require the rapid coordination of multidisciplinary teams, including perfusion, cardiothoracic surgery and intensive care, quick identification of eligible patients, and prompt initiation of ECPR [23].

Overall, this study was informative for our institution, demonstrating that there could be many ECPR-eligible patients. At our centre, future study will be necessary on how to implement an ECPR program to improve the outcomes in this group of patients. Balancing local factors and ECMO availability, our institution may favour a restrictive eligibility strategy to allow for buy-in, program development, purchasing of equipment, training, and simulation. Other centres in Canada may adopt a similar strategy and may conduct similar analyses to anticipate ECPR demand. Additionally, further research into economic analyses, including cost-effectiveness and cost-utility, will likely be required before wide adoption of ECPR programs across Canada [6, 23].

## Limitations

This study had some limitations. First, the retrospective nature of this study may present inherent limitations. Patient information was collected retrospectively from electronic or paper reports, in which desired information may be absent or illegible. Second, EMS records of OHCA were not studied. Therefore, this study only included OHCA patients surviving to presentation to the ED. Some patients died on the scene or prior to ED arrival. Third, the application of ECPR eligibility criteria retrospectively is challenging, without capturing the individual clinicians’ decision-making framework at the time of presentation. Fourth, we were not able to evaluate the effects of ECPR on patient outcomes. In this study, only a limited number of patients had received ECPR. Finally, given the small sample size of this retrospective study, the generalizability of this study is limited. Other centres may want to do further prospective work in this regard.

## Supplementary Information

**Additional file 1.** Supplementary appendix

## Data Availability

The data that support the findings of this study are available on request from the corresponding author, ES, and permission of the Saskatchewan Health Authority. The data are not publicly available due to privacy and confidentiality restrictions from the Saskatchewan Health Authority.

## Abbreviations

CPC: Cerebral performance category
CPR: Cardiopulmonary resuscitation
ECMO: Extracorporeal membrane oxygenation
ECPR: Extracorporeal cardiopulmonary resuscitation
ED: Emergency department
ICU: Intensive care unit
IQR: Interquartile range
OHCA: Out-of-hospital cardiac arrest
PEA: Pulseless electrical activity
ROSC: Return of spontaneous circulation
SD: Standard deviation
UBC: University of British Columbia
UC: University of California
UM: University of Michigan

## Declarations

### Disclaimer

This article does not represent the views of the University of Saskatchewan or the Saskatchewan Health Authority.
