# Supplementary material for: Evaluation of extracorporeal cardiopulmonary resuscitation eligibility criteria for out-of-hospital cardiac arrest patients
Source: BMC Res Notes. 2021 Apr 15;14:139. doi: 10.1186/s13104-021-05564-1 (PMC8051044; doi:10.1186/s13104-021-05564-1)
Supplement: Supplementary file 1 — Additional file 1. Supplementary appendix [file 13104_2021_5564_MOESM1_ESM.docx]

**Additional file 1: Appendix**

**Table S1**. Extracorporeal cardiopulmonary resuscitation eligibility criteria

|  | **University of British Columbia (UBC) criteria** (1) | **University of Michigan (UM) criteria** (2) | **University of California (UC) criteria** (3) | **Restrictive criteria** |
| --- | --- | --- | --- | --- |
| **Trial No.** | NCT02832752 | NCT03065647 |  |  |
| **Inclusion Criteria** | •Age 18-65 years  •Treated by an ALS paramedic  •Witnessed arrest  •Bystander CPR required if initial rhythm is pulseless electrical activity  •Cause of the arrest is presumed to be one of the following: no obvious non-cardiac cause is identified, known overdose of specific drugs (i.e. beta-blocker, calcium channel blocker, tricyclic antidepressants or digoxin), hypothermia (<32°C)  • The patient is pulseless after a minimum of 3 cycles of CPR and intubation. | •Age 18-70 years  •Witnessed arrest or initial shockable rhythm (VT or VF)  •Cause of arrest is presumed to be non-traumatic etiology  •Persistent cardiac arrest after initial cardiac rhythm analysis and shock (if shock is indicated) | •Persistent cardiopulmonary arrest despite traditional resuscitative efforts  •Shock (systolic blood pressure <70 mmHg) refractory to standard therapies | •Age 18-60 years  •Witnessed arrest and the initial rhythm is shockable (VF or VT) |
| **Exclusion Criteria** | •An alternate cause of the arrest is most likely  •DNR status  •Pre-existing comorbidities including congestive heart failure, chronic obstructive pulmonary disease or other significant lung disease, dialysis, liver failure, active malignancy, and pre-existing major neurological deficits  •Asystole as the initial rhythm  •End-tidal CO_2_ less than 10 mmHg at the time of protocol activation | •Sustained return of spontaneous circulation  •Advanced directive indicating DNR or DNI  •Prisoner status  •Pregnancy | • Asystole as the initial rhythm  • Chest compressions not initiated within 10 minutes of cardiac arrest (either by bystanders or EMS personnel)  •Estimated EMS transport time greater than 10 min  •Suspicion of shock due to sepsis or hemorrhage  •Pre-existing severe neurological disease prior to arrest (i.e. traumatic brain injury, stroke, or severe dementia) | • Asystole as the initial rhythm  •Less than 15 minutes of CPR performed without ROSC  •Cardiac arrest due to non-cardiac cause  •Pre-existing comorbidities including congestive heart failure, chronic obstructive pulmonary disease or other significant lung disease, dialysis, liver failure, active malignancy, and pre-existing major neurological deficits |

**Abbreviations:** Advanced cardiovascular life support (ACLS), advanced life support (ALS), carbon dioxide (CO_2_), cardiopulmonary resuscitation (CPR), do not intubate (DNI), do not resuscitation (DNR), return of spontaneous circulation (ROSC), ventricular fibrillation (VF), ventricular tachycardia (VT)

**Table S2**. Demographics, clinical characteristics, interventions, and outcomes of all out-of-hospital cardiac arrest patients, by ECPR eligibility

| **Patient characteristic†** |  | **Any ECPR criteria met**  **(n=35)** | **No ECPR criteria met**  **(n=160)** | **Total**  **(n=200)** | **p-value** | **Missing data (n)** |
| --- | --- | --- | --- | --- | --- | --- |
| ***Clinical characteristics*** | | | | | | |
| Age, median years (IQR) |  | 56 (46-62) | 66 (54-80) | 64 (52-78) | 0.003 | - |
| Male gender, N (%) |  | 28 (80) | 111 (67) | 139 (70) | 0.14 | - |
| Smoker, N (%) |  | 12 (34) | 89 (54) | 101 (51) | 0.09 | - |
| Family history of cardiac event, N (%) |  | 3 (9) | 7 (4) | 10 (5) | 0.29 | 1 |
| Previous ischemic heart disease, N (%) |  | 3 (9) | 32 (20) | 35 (18) | 0.12 | 2 |
| Previous CABG, N (%) |  | 7 (18) | 10 (6) | 17 (9) | 0.19 | 1 |
| Previous PCI, N (%) |  | 0 (0) | 9 (5) | 9 (5) | 0.16 | - |
| Previous AICD, N (%) |  | 0 (0) | 9 (5) | 9 (5) | 0.16 | - |
| Charlson Comorbidity Index, median score (IQR) |  | 0 (0-1) | 1 (0-3) | 1 (0-2) | <0.001 | - |
| ***Details of cardiac arrest*** | | | | | | |
| Initial cardiac rhythm, N (%) | VF | 13 (37) | 41 (25) | 54 (28) | 0.49 | 4 |
|  | VT | 1 (3) | 10 (6) | 11 (6) |  |  |
|  | PEA | 13 (37) | 73 (45) | 86 (44) |  |  |
|  | Asystole | 8 (23) | 37 (23) | 45 (23) |  |  |
| Cause of arrest, N (%) | Ischemic heart disease | 15 (43) | 78 (47) | 93 (47) | 0.48 | - |
|  | Cardiomyopathy | 0 (0) | 4 (2) | 4 (2) |  |  |
|  | PE | 0 (0) | 2 (1) | 2 (1) |  |  |
|  | Hypothermia | 0 | 1 (1) | 1 (1) |  |  |
|  | Drugs | 7 (20) | 20 (12) | 27 (14) |  |  |
|  | Respiratory | 0 (0) | 10 (6) | 10 (5) |  |  |
|  | Unknown | 13 (37) | 50 (30) | 63 (32) |  |  |
| Location of arrest, N (%) | Place of residence | 24 (69) | 102 (62) | 126 (63) | 0.61 | - |
|  | Public place | 10 (29) | 52 (32) | 62 (31) |  |  |
|  | Other | 1 (3) | 11 (7) | 12 (6) |  |  |
| Bystander witnessed, N (%) |  | 32 (91) | 123 (75) | 155 (78) | 0.03 | - |
| First end tidal CO2 measured, median mmHg (IQR) |  | 30 (15-45) | 33 (21-47) | 32 (20-47) | 0.42 | 70 |
| Immediate GCS post arrest, median score (IQR) |  | 3 (3-3) | 3 (3-3) | 3 (3-3) | 0.23 | - |
| APACHEII score in first 24 hours post-arrest, median score (IQR) |  | 33 (28-37) | 32 (27-37) | 33 (28-37) | 0.79 | - |
| ***Laboratory investigations and tests*** | | | | | | |
| Initial arterial blood pH |  | 6.95 (6.76-7.11) | 7.09 (6.89-7.23) | 7.07 (6.87-7.22) | 0.01 | 53 |
| Initial arterial blood PaO_2_, median mmHg |  | 90 (42-195) | 99 (65-172) | 99 (64-180) | 0.82 | 54 |
| Initial arterial blood PaCO_2_, median mmHg |  | 72 (47-95) | 54 (41-72) | 55 (43-77) | 0.08 | 52 |
| Initial HCO_3_, median mmol/L |  | 13 (10-20) | 15 (12-20) | 15 (11-20) | <0.001 | 72 |
| Initial creatinine, median μmol/L |  | 100 (87-144) | 105 (79-164) | 105 (80-159) | 0.93 | 64 |
| Initial lactate, median mmol/L |  | 14.1 (9.9-17) | 9.2 (4.5-13.5) | 10.3 (5.1-14.8) | <0.001 | 56 |
| Initial high sensitivity troponin, median ng/L |  | 21 (6-88) | 32 (12-144) | 32 (11-112) | 0.23 | 72 |
| ST elevation on initial ECG, N (%) |  | 9 (31) | 39 (26) | 48 (27) | 0.60 | 23 |
| ***Interventions during cardiac arrest*** | | | | | | |
| Installation of automated chest compression device, N (%) |  | 34 (97) | 116 (71) | 150 (75) | 0.001 | 1 |
| Defibrillation during initial CPR, N (%) |  | 29 (83) | 94 (57) | 123 (62) | 0.005 | 1 |
| Use of vasopressors during initial CPR, N (%) |  | 34 (97) | 132 (81) | 166 (84) | 0.02 | 3 |

†**Abbreviations:** automated implantable cardioverter-defibrillator (AICD)**,** acute physiology and chronic health evaluation II (APACHEII), coronary artery bypass graft (CABG), coronary care unit (CCU), electrocardiogram (ECG), intensive care unit (ICU), number (N), out-of-hospital cardiac arrest (OHCA), percutaneous intervention (PCI), pulmonary embolism (PE)

**Table S3**. Demographics, clinical characteristics, interventions, and outcomes of all out-of-hospital cardiac arrest patients.

| **Patient characteristic†** |  | **Survivors**  **(n=40)** | **Non-survivors**  **(n=160)** | **Total**  **(n=200)** | **Missing data (n)** |
| --- | --- | --- | --- | --- | --- |
| ***Clinical characteristics*** | | | | | |
| Age, median years (IQR) |  | 57 (44-69) | 65 (54-80) | 64 (52-78) | - |
| Male gender, N (%) |  | 31 (78) | 108 (68) | 139 (70) | - |
| Smoker, N (%) |  | 24 (60) | 77 (48) | 101 (51) | - |
| Family history of cardiac event, N (%) |  | 4 (10) | 6 (4) | 10 (5) | 1 |
| Previous ischemic heart disease, N (%) |  | 9 (23) | 26 (16) | 35 (18) | 2 |
| Previous CABG, N (%) |  | 7 (18) | 10 (6) | 17 (9) | 1 |
| Previous PCI, N (%) |  | 3 (8) | 6 (4) | 9 (5) | - |
| Previous AICD, N (%) |  | 0 (0) | 9 (6) | 9 (5) | - |
| Charlson Comorbidity Index, median score (IQR) |  | 1 (0-3) | 1 (0-2) | 1 (0-2) | - |
| ***Details of cardiac arrest*** | | | | | |
| Initial cardiac rhythm, N (%) | VF | 17 (46) | 37 (23) | 54 (28) | 4 |
|  | VT | 5 (14) | 6 (4) | 11 (6) |  |
|  | PEA | 14 (38) | 72 (45) | 86 (44) |  |
|  | Asystole | 1 (3) | 44 (28) | 45 (23) |  |
| Cause of arrest, N (%) | Ischemic heart disease | 17 (43) | 76 (48) | 93 (47) | - |
|  | Cardiomyopathy | 3 (8) | 1 (1) | 4 (2) |  |
|  | PE | 1 (3) | 1 (1) | 2 (1) |  |
|  | Hypothermia | 0 (0) | 1 (1) | 1 (1) |  |
|  | Drugs | 10 (25) | 17 (11) | 27 (14) |  |
|  | Respiratory | 3 (8) | 7 (4) | 10 (5) |  |
|  | Unknown | 6 (15) | 57 (36) | 63 (32) |  |
| Location of arrest, N (%) | Place of residence | 23 (60) | 103 (64) | 126 (63) | - |
|  | Public place | 12 (30) | 50 (31) | 62 (31) |  |
|  | Other | 5 (13) | 7 (4) | 12 (6) |  |
| Bystander witnessed, N (%) |  | 35 (88) | 120 (75) | 155 (78) | - |
| Duration of CPR performed, median mins (IQR) |  | 5 (3-13) | 40 (20-56) | 30 (12-47) | 54 |
| First end tidal CO2 measured, median mmHg (IQR) |  | 29 (22-35) | 33 (20-51) | 32 (20-47) | 70 |
| Immediate GCS post arrest, median score (IQR) |  | 3 (3-7) | 3 (3-3) | 3 (3-3) | - |
| APACHEII score in first 24 hours post-arrest, median score (IQR) |  | 25 (20-28) | 33 (30-38) | 33 (28-37) | - |
| ***Laboratory investigations and tests*** | | | | | |
| Initial arterial blood pH |  | 7.26 (7.19-7.33) | 6.97 (6.82-7.13) | 7.07 (6.87-7.22) | 53 |
| Initial arterial blood PaO_2_, median mmHg |  | 115 (77-188) | 94 (59-180) | 99 (64-180) | 54 |
| Initial arterial blood PaCO_2_, median mmHg |  | 45 (38-57) | 59 (48-80) | 55 (43-77) | 52 |
| Initial HCO_3_, median mmol/L |  | 20 (17-22) | 13 (10-16) | 15 (11-20) | 72 |
| Initial creatinine, median μmol/L |  | 93 (77-127) | 112 (84-198) | 105 (80-159) | 64 |
| Initial lactate, median mmol/L |  | 4.2 (2.7-6.4) | 12.5 (8.7-15.0) | 10.3 (5.1-14.8) | 56 |
| Initial high sensitivity troponin, median ng/L |  | 29 (13-144) | 32 (9-111) | 32 (11-112) | 72 |
| ST elevation on initial ECG, N (%) |  | 14 (35) | 34 (25) | 48 (27) | 23 |
| ***Interventions*** | | | | | |
| Installation of automated chest compression device, N (%) |  | 18 (45) | 132 (83) | 150 (75) | 1 |
| Defibrillation during initial CPR, N (%) |  | 23 (58) | 100 (63) | 123 (62) | 1 |
| Use of vasopressors during initial CPR, N (%) |  | 20 (50) | 146 (93) | 166 (84) | 3 |
| Coronary angiography, N (%) |  | 19 (48) | 14 (9) | 33 (17) | - |
| PCI, N (%) |  | 8 (20) | 9 (6) | 17 (9) | - |
| CABG, N (%) |  | 5 (13) | 0 (0) | 5 (3) | - |
| ECPR, N (%) |  | 1 (3) | 1 (1) | 2 (1) | 2 |
|  |  |  |  |  |  |
| ***Outcomes*** | | | | | |
| ICU or CCU admission, N (%) |  | 26 (67) | 47 (29) | 73 (37) | 1 |
| Complications, N (%) | Circulatory shock | 9 (23) | 40 (25) | 49 (25) | - |
|  | Need for RRT | 5 (13) | 9 (6) | 14 (7) | - |
|  | Stroke | 1 (3) | 2 (1) | 3 (2) | 1 |
|  | Intracerebral hemorrhage | 1 (3) | 1 (1) | 2 (1) | - |
| Hospital length of stay, median days (IQR) |  | 9 (5-16) | 0 (0-1) | 0 (0-4) | 2 |
| ICU length of stay, median days (IQR) |  | 3 (0-5) | 0 (0-1) | 0 (0-2) | 2 |
| Cerebral Performance Category at discharge, median score (IQR) |  | 2 (1-2) | 5 (5-5) | 5 (5-5) | - |
| Cerebral Performance Category at discharge, N (%) | 1 | 18 (45) | 0 (0) | 18 (9) | - |
|  | 2 | 15 (38) | 0 (0) | 15 (8) |  |
|  | 3 | 6 (15) | 0 (0) | 6 (3) |  |
|  | 4 | 1 (2) | 0 (0) | 1 (0) |  |
|  | 5 | 0 (0) | 160 (100) | 160 (80) |  |
| In-hospital mortality, N (%) |  | 0 (0) | 160 (100) | 160 (80) | - |

†**Abbreviations:** automated implantable cardioverter-defibrillator (AICD)**,** acute physiology and chronic health evaluation II (APACHEII), coronary artery bypass graft (CABG), coronary care unit (CCU), electrocardiogram (ECG), intensive care unit (ICU), number (N), out-of-hospital cardiac arrest (OHCA), percutaneous intervention (PCI), pulmonary embolism (PE)

**Additional Appendix References**

1. Grunau B. BC ECPR Trial for Out-of-Hospital Cardiac Arrest (Clinicaltrials.gov Identifier NCT02832752) [Internet]. 2017. Available from: https://clinicaltrials.gov/ct2/show/NCT02832752

2. Neumar R. ECPR for Refractory Out-Of-Hospital Cardiac Arrest (EROCA) (Clinicaltrials.gov Identifier NCT03065647) [Internet]. 2017. Available from: https://clinicaltrials.gov/ct2/show/NCT03065647

3. Bellezzo JM, Shinar Z, Davis DP, Jaski BE, Chillcott S, Stahovich M, et al. Emergency physician-initiated extracorporeal cardiopulmonary resuscitation. Resuscitation. 2012;83(8):966–70.
